# FarGen: Elucidating the distribution of coding variants in the isolated population of the Faroe Islands

**DOI:** 10.1038/s41431-022-01227-2

**Published:** 2022-11-21

**Authors:** Ólavur Mortensen, Elisabet Thomsen, Leivur N. Lydersen, Katrin D. Apol, Pál Weihe, Bjarni á Steig, Guðrið Andorsdóttir, Thomas D. Als, Noomi O. Gregersen

**Affiliations:** 1 The Genetic Biobank of the Faroe Islands, Tórshavn, Faroe Islands; 2 Department of Occupational Medicine and Public Health, National Hospital of the Faroe Islands Tórshavn, Tórshavn, Faroe Islands; 3 Medical Department, National Hospital of the Faroe Islands, Tórshavn, Faroe Islands; 4 Centre of Health Science, Faculty of Health, University of the Faroe Islands, Tórshavn, Faroe Islands; 5 Department of Biomedicine, Aarhus University, Aarhus, Denmark; 6 The Lundbeck Foundation Initiative for Integrative Psychiatric Research, iPSYCH, Aarhus, Denmark; 7 Center for Genomics and Personalized Medicine, Aarhus, Denmark

**Keywords:** Genetic variation, Clinical genetics

## Abstract

Here we present results from FarGen Phase I exomes. This dataset is based on the FarGen cohort, which consists of 1,541 individuals from the isolated population of the Faroe Islands. The purpose of this cohort is to serve as a reference catalog of coding variants, and to conduct population genetic studies to better understand the genetic contribution to various diseases in the Faroese population. The first whole-exome data set comprise 465 individuals and a total of 148,267 genetic variants were discovered. Principle Component Analysis indicates that the population is isolated and weakly structured. The distribution of variants in various functional classes was compared with populations in the gnomAD dataset; the results indicated that the proportions were consistent across the cohorts, but probably due to a small sample size, the FarGen dataset contained relatively few rare variants. We identified 19 variants that are classified as pathogenic or likely pathogenic in ClinVar; several of these variants are associated with monogenetic diseases with increased prevalence in the Faroe Islands. The results support previous studies, which indicate that the Faroe Islands is an isolated and weakly structured population. Future studies may elucidate the significance of the 19 pathogenic variants that were identified. The FarGen Phase I dataset is an important step for genetic research in the Faroese population, and the next phase of FarGen will increase the sample size and broaden the scope.

## Introduction

In recent years, a number of large-scale whole-exome sequencing projects have been conducted. The appeal of whole-exome sequencing is the ability to gain insight into biologically relevant and clinically actionable variants. Furthermore, we may gain insight into rare and functionally important variants in a cost-effective manner. The power of whole-exome sequencing is demonstrated by e.g., Van Hout et al. [1] who showed a 14-fold increase of loss-of-function variants when comparing 49,960 exomes with imputed microarray data using largely overlapping samples. In a different study based on 50,726 exomes, Dewey et al. [2] discovered deleterious variants in 3.5% of the participants, across 76 clinically actionable genes.

Isolated populations may present interesting opportunities for research into the genetic architecture of hereditary diseases. The Faroe Islands, present such a population. Based on mtDNA sequence data Als et al. [3] showed that the Faroe Islands are among the most isolated populations in the North Atlantic. The analyses indicated low genetic diversity and a small effective population size [3, 4]. This is likely due to several bottlenecks followed by population expansion, in combination with extended periods of isolation [4]. Isolation, combined with the small effective population size, may have led to substantial genetic drift and loss of genetic diversity; this in turn may have led to an enrichment of some pathogenic variants and complete loss of others [5–7]. Several hereditary diseases have significantly increased prevalence in the Faroe Islands. This includes diseases with simple genetic architecture, such as cystic fibrosis (CF) [6], carnitine transporter deficiency [8], 3-methylcrotonyl-CoA carboxylase deficiency (3-MCCD) [9], and mitochondrial encephalomyopathy (MNGIE) [10], and complex diseases like inflammatory bowel disease [11]. This skewed morbidity is likely caused by genetic drift.

The Faroe Genome Project, FarGen, is an initiative to build cohorts and infrastructure for genetic research in the isolated population of the Faroe Islands. In the first phase of the project, we collected blood samples from 1541 Faroese individuals, corresponding to 3% of the total population [12]. The purpose of this cohort is to serve as controls for association studies, to build a catalog of genetic variants, and for population genetic studies in general.

Here we present preliminary results from 465 exomes from FarGen Phase I. First and foremost, we present a catalog of coding variants to serve as a preliminary reference panel. Additionally, we investigate population structure, the distribution of variants by functional class and impact, and identify variants classified as pathogenic or likely pathogenic in ClinVar [13]. Throughout, we compare the FarGen dataset with data in the Genome Aggregation Database (gnomAD) [14].

## Subjects and methods

### The FarGen cohort

The FarGen cohort comprise 1541 individuals. The recruitment procedures, socio-demographics, availability of the bioresources, DNA extraction, DNA barcoding using the 10x Genomics-Chromium controller, library construction, and sequencing has been described previously [12, 15, 16]. In short, inclusion criteria were: 1) participants had to be 18 years or older, 2) had to live in the Faroe Islands or be of Faroese descent, and 3) had to join the project voluntarily. The participants reported their health/disease status. Exomes were enriched using the SureSelectXT Human All Exon V6 (Agilent Technologies, Santa Clara, CA, USA), and linked-read sequenced with a mean depth of 50x, using the Illumina NextSeq500 platform at the Research Park iNOVA, Faroe Islands.

Here we have included whole-exome sequencing data from 536 participants. The lineages of the participants were investigated using the Multi-Generation Registry of the Faroe Islands (https://biobank.fo/english/). A heuristic was used to ensure that all individuals to some degree have ancestry within the Faroese population. Firstly, only individuals that are recorded in the Multi-Generation Registry (MGR) are included. Secondly, individuals with ancestry lineages of length between 0 and 2 in the MGR were removed. The 473 remaining individuals had lineages of length between 6 and 27 generations.

### LinkSeq pipeline

For the bioinformatic analyses we developed a set of pipelines, dubbed LinkSeq, to process linked-read sequencing data, with an emphasis on population-based variant discovery. Details on these pipelines can be found at the GitHub repository: https://github.com/fargenfo/linkseq. In short, variants were called and filtered, then datasets were merged and subsequently filtered jointly. A variant is “present” in a dataset/population if it’s polymorphic in that dataset. Since singletons are removed, this essentially means that the allele count is larger than 1.

LinkSeq was developed to process the linked-read data in a manner that is suitable for population-based analyses and adapts the GATK Best Practices guidelines [17] to linked-reads [16], applying available open-source tools developed for linked-reads. One of the primary benefits of linked-reads is the ability to unambiguously align reads in homologous regions of the genome [18, 19]. LinkSeq thus combines the power of linked-reads with the benefits of population-based variant discovery in GATK. Other analysis performed in this project are described in this GitHub repository: https://github.com/olavurmortensen/fargen-1-exome/tree/master/notebooks/main/html.

### Base calling and read trimming

Bases were called and samples were demultiplexed simultaneously using bcl2fastq v2.20, using the quadruple redundant 10x Genomics octamer index barcodes. The adapter sequences on the 3’end were trimmed using BBtools v37.62, poly-G tails were trimmed using fastp v0.20, and bases with poor quality were trimmed using Sickle v1.33. An algorithm was developed in-house to trim reads contaminated with 10x Genomics barcodes. This barcode is 16 bp long and is situated in the beginning of read 1. If the insert is shorter than the read length (150 bp), then read 2 will include parts of this barcode, and therefore, this barcode is trimmed from read 2.

### Read mapping

The sequencing reads were aligned to the GRCh38/hg38 human reference genome using the EMA v0.6 aligner [18], using 500 barcode bins. Reads with no barcode were aligned using BWA v0.7 [20]. The 500 barcode bins, together with the no-barcode bin, were subsequently merged and coordinate sorted. Duplicate reads were marked, all reads were indexed, and Base Quality Score Recalibration (BQSR) was applied using GATK v4.1, supplying known sites via dbSNP build 138 [21]. Samples with average coverage under 5x were excluded.

### Variant discovery and annotation

Variant discovery was performed in accordance with the GATK Best Practices for germline short-variant discovery [14]. Variants were called using GATK v4.1, using the GRCh38 reference genome, forcing calls at dbSNP 138 sites, while restricting calls to target regions (SureSelectXT Human All Exon v6). The variant calling was done in two stages, first discovering variable sites with GATK HaplotypeCaller and then joint genotyping using GATK GenotypeGVCFs. Genotyping was restricted to variants with quality score (QUAL) above 200.

SNPs and indels were filtered separately using GATK v4.1 Variant Quality Score Recalibration (VQSR). A maximum of 6 Gaussians were used for SNPs and 4 for indels, and a truth sensitivity level of 50.0 (see the LinkSeq pipeline code repository for details). The genotype calls were refined using GATK v4.1 CalculateGenotypePosteriors, updating the probability of observing the genotypes in the call-set, and updating the genotypes based on the posterior probability. Invariant sites and unused alternate alleles that did not pass QC were removed after this step. The variants were annotated with RefSNP numbers from dbSNP 138 using GATK v4.1 VariantAnnotator. SnpEff v4.3 was used to annotate the functional effect and impact of variants.

### Filtering

The following filters were applied to the joint genotyped variants, to create a dataset containing high quality variants. Variants in the 50 to 100 VQSR tranches were removed. The following hard-filters as specified in GATK and VCF tools were applied to the *SNPs*: QD < 2.0, SOR > 3.0, FS > 60.0, MQ < 40.0, MQRankSum < −12.5, ReadPosRankSum < −8.0. The following hard-filters were applied to the *indels*: QD < 2.0, FS > 200.0, ReadPosRankSum < −20.0. Genotypes with low genotype quality (GC) [17] were discarded, using GC < 20 for SNPs and GC < 40 for indels. Heterozygous genotypes with allelic balance (AB) less than 0.25 were discarded. Variants failing test for Hardy–Weinberg Proportions were removed, using *p* < 1e-9 for SNPs and *p* < 1e-6 for indels. Singletons were removed due to overall bad quality. Four samples were discarded due to higher heterozygote/homozygote rate.

### Population structure

**Fig. 1:**
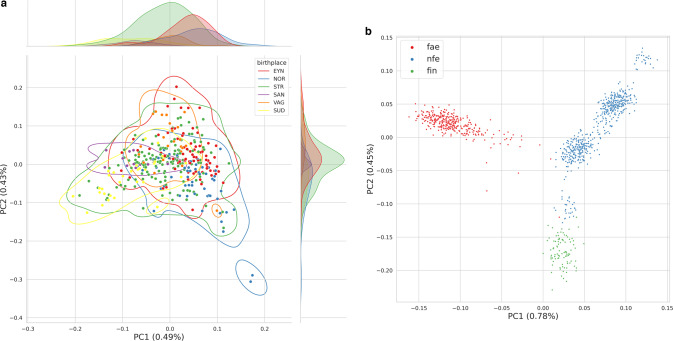
The Principal Component Analysis plots shows the first and second principal components, and percent of variance explained by each principal component is noted in parenthesis on the axes. **a** shows the FarGen dataset, where the labels show the samples’ region of birth, abbreviated as follows: NOR (Norðoyggjar), EYN (Eysturoy og Norðstreymoy), STR (Suðurstreymoy), VAG (Vágar og Mykines), SAN (Sandoy, Skúvoy, Stóra Dímun), and SUD (Suðuroy). Contours of kernel densities are shown for each label, and the marginalized densities are shown in the top and right of the plot. **b** shows the PCA of the *merged genotype dataset*, where the labels show the origin of the samples, abbreviated as follows: fae (Faroe Islands), fin (Finland), nfe (non-Finnish European).

Principal Component Analysis (PCA) was used to investigate population structure. First, variants were linkage disequilibrium (LD) pruned with a window size of 500,000 bp and *r*^2^ < 0.2. Next, closely related individuals were discarded from PCA analysis. The realized kinship coefficients between all individuals were computed using the PC-Relate method [22, 23]. A greedy algorithm was used to prune individuals with kinship coefficient above 1/16. Finally, we computed the Hardy-Weinberg equilibrium (HWE) normalized PCA of the Generalized Relationship Matrix (GRM) using Hail 0.2 [23]. Four samples were discarded due to being outliers in the first principal component. This left 465 samples for a second and final PCA (Fig. 1a). Further, to visualize potential geographic population structure, we stratify the samples by birthplace. We defined six regions (see Fig. 1a) of the Faroe Islands based on differences in dialect, using isoglosses as defined in Thráinsson et al. [24]. Here, we apply the principle that genetic differentiation and dialect are closely connected [25]. Further, to compare structure in the Faroese population with other European populations we performed a PCA on a *merged genotype dataset* (see description of the *merged genotype dataset* in the next section).

### Merged genotype dataset

The FarGen genotypes were merged with genotypes in gnomAD v3.1. Specifically, the HGDP+1KG callset [14, 26] was used, and we shall refer to this dataset as the *merged genotype dataset*.

Before merging the data, we filtered the gnomAD variants as done in Karczewski et al. 2020 [14]. We filtered the gnomAD sites to contain only those within the exome targets used in FarGen. Indels were discarded. The datasets were merged such that only *sites present in both* datasets were included.

### Merged sites dataset

The FarGen sites were merged with the gnomAD v2.1.1 exome sites data. We shall refer to this dataset as the *merged sites dataset*. We applied the same filters to the gnomAD variants as described in the previous section. A union of the FarGen and gnomAD datasets was created, such that the resulting dataset contained all sites in both datasets. Subsequently, variants outside the calling regions of either dataset were discarded. Variants in repetitive regions of the genome were removed, RepeatMasker v3.0.1 was used to find such regions (http://www.repeatmasker.org.). Finally, the variants in the merged sites dataset were annotated with functional impact and effect using SnpEff v4.3.

### Imputing sex

We imputed the sex of the samples based on the inbreeding coefficient (F) on the X chromosome. Samples with *F* < 0.4 are imputed as female while samples with *F* > 0.4 are imputed as male.

### Folded site-frequency spectrum

The Folded Site-Frequency Spectrum (FSFS) were computed for variants in the FarGen dataset, and the *merged sites dataset* as follows: Let *f*_*i*_ be the *site-frequency* of frequency bin *i*. The FSFS is calculated as *f*^∗^_*i*_ = *f*_*i*_ + *f*_*n−i*_, where *n* is the number of bins. For example, if we have 10 bins, then *f*_1_ corresponds to frequencies between 0 and 0.1, and *f*^∗^_1_ = *f*_1_ + *f*_10_.

Before FSFS computation the variants in the FarGen dataset were stratified by effect, while the variants in the *merged sites dataset* were stratified by population (Faroese, Finnish, non-Finnish North-Western European, and *total* gnomAD populations).

### Pathogenic variants

The variants in the FarGen dataset were annotated with clinical significance from ClinVar [13] (accessed 7th of November 2021). Variants classified as “pathogenic” or “likely pathogenic” were included and annotated with allele frequencies from gnomAD v2.1.1 exome sites data. We also checked whether any of the identified “pathogenic” or “likely pathogenic” variants are clinically actionable, according to the Geisinger 76 gene list [2], which includes the 56 genes identified in the American College of Medical Genetics and Genomics (ACMG) guidelines for reporting of clinically actionable genetic findings [27].

## Results

A total of 911,912 variants were called in 473 samples and after all filters, such as read coverage, genotype call rate, allelic balance, heterozygosity rate, HWE, and missing genotypes were applied, 148,267 variants and 465 samples remained in the final dataset (see exclusion filters in Supplementary Fig. 1). About 84% of the variants were SNPs and 16% were indels. The average genotype depth was 38 and the average allelic balance for heterozygotes was 0.49. The average heterozygote/homozygote rate was 0.26, and the transition/transversion rate was 2.5. Of the 465 samples, 275 were females and 190 were males. This result was obtained by imputing sex using the inbreeding coefficient F on the X chromosome, as described above. The females had an inbreeding coefficient of F = −0.15 ± 0.22 and the males had an inbreeding coefficient of F = 0.91 ± 0.06. No individuals were removed due to sex check.

**Fig. 2:**
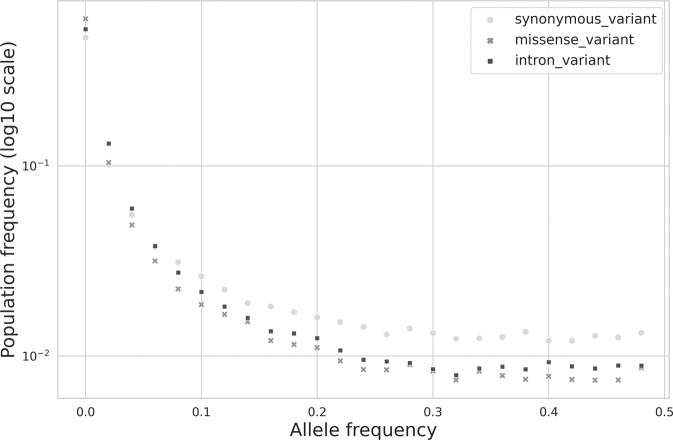
The figure shows the Folded Site-Frequency Spectrum (FSFS) in the FarGen dataset, computed as described in the methods section. The variants are stratified by effect, as annotated with SnpEff before the FSFS computation. Y-axis is on a log-10 scale.

The FSFS for variants stratified by effect in the FarGen dataset is shown in Fig. 2. Here the FSFS were computed on the *unfiltered* data from 473 samples. In this FSFS, 50 frequency bins were used, so that the first bin represents variants in the 0–0.02 frequency range. Fifty-nine percent of the missense variants have a frequency under 0.02, while this number is 52% for intron variants and 47% for synonymous variants. We see that there is an over-representation of missense variants in the low frequency end of the spectrum, and an under-representation in the high frequency end of the spectrum.

### Population structure

The PCA of the FarGen dataset is shown in Fig. 1a. After LD pruning, relatedness pruning, and discarding variants with a minor allele frequency < 0.01 (MAF), 47,253 variants and 382 samples remained in the dataset. As shown in Fig. 1a, principal components 1 and 2 explain 0.49% and 0.43% of the total variance, respectively. The PCA plot of the FarGen dataset indicates some degree of weak genetic gradients across the six geographical regions. However, the contours of the densities, estimated via kernel density estimation, overlap substantially. We also see this in the marginal densities, indicated in the top and right of the plot. The capital region, labeled “STR” and indicated in green, seems to cover the entire plot, overlapping with all the other regions.

The PCA (PCs 1 and 2) of the *merged genotype dataset* is shown in Fig. 1b, and was performed as in the previous section, only now discarding variants with MAF < 0.05. The final *merged genotype dataset*, after all filters, contained 27,590 variants and 4,411 samples. The PCA was computed on the European populations present in the gnomAD and FarGen dataset, labeled by Faroe Islands (red), Finland (green), and non-Finnish European (blue). PCs 1 and 2 explain 0.78% and 0.45% of the total variance, respectively. We see that the Faroese and Finnish populations form distinct clusters that are separate from the rest of the European populations.

### Comparison with gnomAD

We compare the Faroese population to other populations in the *merged sites dataset*. Variants with allele count larger than one (i.e., non-singletons) were considered to be *in* a population. A total of 14,946,048 variants were present in either the Faroese or the *total* gnomAD population, while 14,886,696 were private to gnomAD across all sub populations, only 55 were private to the Faroese population. This left 59,297 in the intersection, variants present in both populations. This means that almost all variants discovered in the FarGen dataset were present in the *total* gnomAD dataset. Note that this analysis is restricted to the exome targets of both datasets, and to non-repetitive regions of the genome, which is why the number of variants in the FarGen dataset is smaller than stated earlier.

**Fig. 3:**
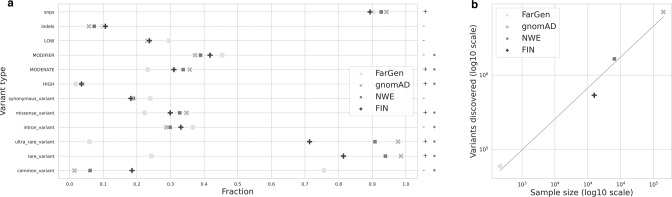
Distribution of genetic variants. **a** Fraction of variants belonging to various classes stratified by population. Populations are labeled as follows: FarGen (Faroe Islands), gnomAD (all samples in gnomAD), NWE (non-Finnish North-Western European), FIN (Finland). Least-Squares regression was performed between the fraction and the log of sample size for all variant types; the sign of the slope is shown on the right-hand side and significance (*p* < 0.05) is noted by an asterisk (*). **b** Number of samples and variants discovered in the same populations as in a. Both axes are on a log10 scale, and a linear regression line is shown in the plot.

Figure 3a shows the fraction of variants belonging to various classes of variants. The data is stratified by population, where we have included the Faroese population, the total gnomAD population, non-Finnish north-western Europeans (NWE), and the Finnish population. Figure 3b shows the sample sizes and variants discovered (non-singletons) in these populations, on a log-log scale.

A pattern emerges in Fig. 3a. These variables, such as the fraction of missense variants, are proportional to the number of samples in the cohort. For example, if we look at the fraction of missense variants, we see that the Faroese population has the lowest fraction followed by the Finnish, NWE, and total gnomAD populations. If we look at the fraction of intron variants, we notice the exact opposite pattern. Note as well in Fig. 3a that ultra-rare and rare variants are under-represented in the small sample sizes, while common variants are over-represented. If we compare this to Fig. 3b, we see that the number of variants discovered is directly proportional to the number of samples in the cohort, as expected, but indicating that the FarGen WES data results in the expected number of called variants and therefore can be considered of high quality.

We tested the relationship between the fraction of variants and the log of sample size for all variant classes via Least-Squares regression. The sign of the slope and the significance is indicated on the right-hand side of Fig. 3a. With a few exceptions, this relationship is statistically significant at *p* < 0.05. In other words, there is an exponential relationship between the sample size and variables such as the fraction of missense variants or rare variants.

**Fig. 4:**
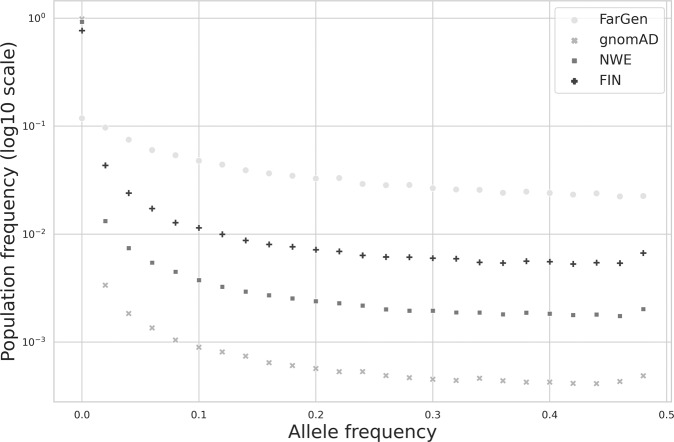
Folded Site-Frequency Spectrum (FSFS), calculated from the *merged sites dataset* between the Faroese (FarGen), Finnish (FIN), non-Finnish North-Western European (NWE), and *total* gnomAD populations (gnomAD). Y-axis is on a log-10 scale. The variants are stratified by population prior to FSFS computation.

Figure 4 shows the FSFS, computed on the *merged sites dataset*, stratified by population. Note, to reduce the number of false positive calls, singletons were ignored. To compare different samples of different size in the same FSFS plot, we used 50 frequency bins; the first bin represents variants in the 0–0.02 frequency range. We see that while only about 12% of the variants in the FarGen dataset have allele frequency less than 0.02, this number is 77%, 93% and 98% for the Finnish, NWE and total gnomAD populations, respectively. In general, the FarGen dataset has more high frequency and fewer low frequency variants. Moreover, if we order the datasets by proportion of low frequency from lowest to highest, it is FarGen, Finnish, NWE and total gnomAD, we see the same pattern as seen in Fig. 3a, b.

### Pathogenic variants

Using the ClinVar pathogenetic classification we identified 19 pathogenic or likely pathogenic variants within 19 different genes (Table 1). These include variants within disease-causing genes for common monogenetic diseases in the Faroese population, such as CTD (*SLC22A5*) [8], as well as complex disease, such as Parkinson´s disease (*LRPPRC*) [28]. The variants comprise 6 frameshift, 11 missense, 2 splice donor variants. Table 1 shows MAF for the 19 variants in the FarGen dataset, *total* gnomAD population (gnomAD), and the non-Finnish north-western European population in gnomAD (gnomAD NWE). Across the 19 variants MAF ranged from 0.379 to 0.005 in FarGen, 0.448 to 0.00001 in gnomAD, and 0.458 to 0.00003 in gnomAD NWE. Thirteen out of 19 variants have a MAF > 0.01 in the FarGen dataset, while 3 and 4 out of the 19 variants have a MAF > 0.01 in gnomAD and gnomAD NEW, respectively. In comparison, 7 of the variants with a MAF > 0.01 in the FarGen dataset have a MAF < 0.0001 in gnomAD. The most skewed frequency is seen for the frameshift variant rs774996406 in the platelet-type bleeding disorder type 18 associated gene *RASGRP2* [29], which has a 3 × 10^3^ fold higher frequency in the FarGen dataset compared to gnomAD. None of the identified pathogenic variants were within the Geisinger 76 genes; [2] hence, the 19 pathogenic variants identified in the FarGen dataset are not clinically actionable according to the ACMG guidelines [27].

**Table 1:** The 19 pathogenic or likely pathogenic variants identified in the FarGen dataset.

						Minor allele frequency		
Gene	Genomic pos.	dbSNP	Nucleotide	Protein	Molecular consequence	FarGen	gnomAD	gnomAD NWE
MCCC1	chr3:183037285	rs727504002	c.1526delG	p.Cys509fs	Frameshift	0.024	1.0E-05	0
SUCLA2	chr13:47988540	rs113994161	c.534 + 1 G > A	-	Splice donor	0.034	1.0E-05	0
CD46	chr1:207761338	rs202071781	c.565 T > G	p.Tyr189Asp	Missense	0.003	2.0E-06	0
SLC22A5	chr5:132370067	rs72552725	c.95 A > G	p.Asn32Ser	Missense	0.045	2.0E-06	0
RASGRP2	chr11:64730127	rs774996406	c.1479dupG	p.Arg494fs	Frameshift	0.129	4.0E-06	2.7E-05
LRPPRC	chr2:43918025	rs769022521	c.3146_3147delAA	p.Lys1049fs	Frameshift	0.019	4.0E-06	4.8E-05
COL1A1	chr17:50188631	rs72653177	c.3106 C > T	p.Arg1036Cys	Missense	0.021	6.0E-06	0
FANCE	chr6:35457937	rs749898067	c.929dupC	p.Val311fs	Frameshift	0.051	9.0E-06	2.0E-04
SPG7	chr16:89531962	rs760818649	c.1053dupC	p.Gly352fs	Frameshift	0.053	1.3E-04	4.0E-04
EXOSC3	chr9:37783993	rs141138948	c.395 A > C	p.Asp132Ala	Missense	0.002	4.1E-04	9.0E-04
C6	chr5:41149933	rs76202909	c.2381 + 2 T > C	-	Splice donor	0.005	0.002	0.004
ACSF3	chr16:89154148	rs141090143	c.1672C > T	p.Arg558Trp	Missense	0.018	0.003	0.006
KIAA0586	chr14:58432438	rs534542684	c.428delG	p.Arg143fs	Frameshift	0.004	0.003	0.004
PADI3	chr1:17270928	rs144080386	c.881 C > T	p.Ala294Val	Missense	0.004	0.007	0.009
CFTR	chr7:117559590	rs1801178	c.1521_1523del	p.Phe508del	Missense	0.028	0.007	0.016
BCHE	chr3:165830741	rs1799807	c.293 A > G	p.Asp98Gly	Missense	0.034	0.012	0.020
MBL2	chr10:52771482	rs5030737	c.154 C > T	p.Arg52Cys	Missense	0.054	0.056	0.071
IL4R	chr16:27344882	rs1805010	c.223 A > G	p.Ile75Val	Missense	0.379	0.448	0.458
MAD1L1	chr7:2225526	rs121908982	c.175 C > T	p.Arg59Cys	Missense	0.004	0.003	0.006

The table includes the associated gene (abbreviates), genomic position (hg38), RSID. from dbSNP, nucleotide change, protein change, molecular consequence, and minor allele frequency (MAF) in FarGen, total gnomAD population (gnomAD), and the non-Finnish. north-western European population in gnomAD (gnomAD NWE). We used the ClinVar classification of pathogenicity and allele frequencies from gnomAD v2.1.1 exome sites data.

## Discussion

Here we presented coding variants discovered in the FarGen cohort comprising individuals descending from the Faroe Islands. We have investigated population structure, both within the population and in relation to other European populations. We investigated how variants of various functional classes and impact were distributed in the FarGen and the gnomAD datasets and juxtaposed this with the frequency spectra of variants. Finally, variants that are known to be pathogenic or likely pathogenic in ClinVar were identified and allele frequencies in different populations were compared.

The results show that there is almost no geographical structure in the Faroese population (Fig. 1a), as all the regions overlap in the first and second principal components, rather than forming distinct clusters. There is, however, a tendency for very weak genetical gradients towards the more remote parts of the Faroe Islands in principal component 1 and 2, but the Faroese population appear to be rather homogenous in general. Previously Als et al. 2014 [30] applied Discriminant Analysis of Principal Components (DAPC) to assess cryptic relatedness and structure in a small sample (*n* = 193) of the Faroese population using low coverage whole-genome-sequence data. The DAPC method, using grandparental island-of-origin as group priors, may overestimate population structure, but still Als et al. concluded that the population is weakly structured according to geography. The current study, using a more conservative approach and analyzing genetic variation limited to the exome, showed that the Faroese population is genetically rather homogeneous. Whether this relatively subtle difference between conclusions of the two studies is caused by differences in applied method, difference in sampling across islands or the specific genomic regions (WGS vs. WES) studied remains unresolved. However, cryptic relatedness and even subtle population structure can lead to false positives in genetic association studies, particularly in founder populations that have experienced a recent rapid growth [31]. Therefore, despite the weak population structure, it is important to take it into account e.g., in association studies [32, 33], but this can relatively easily be corrected for by including the first principal components as covariates. A nationwide study in the genetically related but less isolated Danish population also revealed remarkable genetic homogeneity and concluded that this could render population structure a lesser concern for future genetic associations studies in Denmark [34].

The PCA results of the FarGen and gnomAD data (Fig. 1b) suggest that the Faroese population is substantially different from the general European population. This result is consistent with the previous report from analysing different North-Atlantic populations applying mtDNA sequencing data, that the Faroes Islands are among the most isolated populations in the North-Atlantic [3].

The FarGen dataset shows an under-representation of potentially deleterious variants (Fig. 3a). This result was consistent for all variant effect and impact annotations, although in some cases it was not statistically significant. It may be tempting to say that this is due isolation, as both the Faroese and Finnish populations are considered being isolated and showed the same pattern in Fig. 3a. This may also be due to samples size, as we see that fewer rare variants are discovered in the smaller cohorts, as well as deleterious variants tend to be young and rare due to selective pressures, which is more evident in smaller populations. However, it is likely due to a combination of population size, sample size, and differences in the way ultra-rare variants are called in the different studies.

A total of 19 pathogenic or likely pathogenic variants were identified in the FarGen dataset (Table 1). Most of the pathogenic variants i.e., 14 out of 19 showed an elevated frequency in the Faroese population in comparison with the gnomAD populations. As expected, we identified several previously identified pathogenic variants associated with common diseases in the Faroese population, such as rs72552725 for CTD [35], rs1801178 for CF [6], rs727504002 for 3-MCCD [9], and rs113994161 MNGIE [10]. Other, pathogenic variants with an elevated frequency in the Faroese population compared to gnomAD were within genes associated with cancer (*FANCE* and *CD46*) [36, 37], Parkinson´s disease (*LRPPRC*) [38], Ehlers-Danlos syndrome (*COL1A1*) [39], platelet-type bleeding disorder type 18 (*RASGRP2*) [30], butyrylcholinesterase deficiency (*BCHE*) [40], spastic paraplegia type 7 (*SPG7*) [41], pontocerebellar hypoplasia (*EXOSC3*) [42], complement component-6 deficiency (*C6*) [43], and combined malonic and methylmalonic aciduria (*ACSF3*) [44]. To the best of our knowledge Parkinson´s disease is the only one of these diseases with an increased prevalence in the Faroese population [29]. The *LRPPRC* gene has not yet been associated with patients with Parkinson´s disease from the Faroe Islands and may be considered as a candidate gene in future studies. Further, more research must be conducted regarding the prevalence of these diseases and the impact of the pathogenic variants; especially concerning the very high frequency of the frameshift variant rs774996406 and its association with platelet-type bleeding disorder type 18. However, there are no records for an elevated frequency of platelet-type bleeding disorder type 18 neither in the FarGen dataset nor in the Faroese population in general, therefore, it is unlikely that this variant is disease causing in the Faroese population. We also identified pathogenic variants with the same or lower frequency in FarGen compared to gnomAD. These comprise variants within genes associated with various types of human cancer (*MAD1L1*) [45], Joubert syndrome 23 (*KIAA0586*) [46], hair disorders (*PADI3*) [47], mannose-binding lectin deficiency (*MBL2*) [48], and multiple sclerosis (*IL4R*) [49]. Of these diseases we only have records on an increased prevalence of multiple sclerosis in the Faroese population [50], therefore future studies may explore the prevalence of these diseases as well as the impact of the identified pathogenic variants. To merged genotypic data with phenotypic data is not in the scope of this study. However, looking into our phenotypic data we can verify that CTD (*SLC22A5*), Parkinson´s disease (*LRPPRC*), multiple sclerosis (*IL4R*), 3-MCCD (*MCCC1*), and different cancer types (*FANCE, MAD1L1 and CD46*) have been reported by the participants. Future perspective will be to merge phenotypes data with genotype data to verify the associations and to report possible incidental findings.

In general, the FarGen dataset was comparable to the gnomAD dataset, and the results were as expected. This tells us that the data quality is high, and we can therefore confidently use it as a reference dataset.

In this project 1% of the Faroese population were sequenced. While this is a commendable achievement, we have seen that there is much to be gained by increasing sample size. By increasing the sample size, we will be able to capture more rare variants and thereby most likely also more deleterious variants.

Whole-exome sequencing is a powerful tool to get insight into protein-altering variants in a cost-effective manner. However, to perform demographic analyses, such as historical effective population size or ancestry inference, and thorough comparison with sequence data from other population whole-genome sequencing is necessary. The next phase of FarGen will address this by sequencing more Faroese individuals using low-pass whole-genome sequencing, which could be useful as a local imputation reference in future GWAS, focusing on both common and rare variants also located outside the exome.

Finally, the FarGen Phase I exome dataset will prove useful in future genetic studies in the Faroe Islands. The catalog of coding variants will serve as a reference for allelic frequency, which may be used in pilot studies as well as in association studies.

## Supplementary information

Supplementary figure 1

## Data Availability

Data from this study is stored at the Genetic Biobank of the Faroe Islands. Data is available for research up on participants’ re-consent. Researchers will be granted access to de-identified genetic-data and meta-data provided that the project protocol has been approved by the Faroese Scientific Ethical Committee and a template material/data transfer agreement has been signed with the Genetic Biobank of the Faroe Islands in compliance with GDPR (see reference [15]).

## Code availability

LinkSeq pipeline: https://github.com/fargenfo/linkseq

Code to reproduce results: https://github.com/olavurmortensen/fargen-1-exome/tree/master/notebooks/main/html
