# Supplementary material for: FarGen: Elucidating the distribution of coding variants in the isolated population of the Faroe Islands
Source: Eur J Hum Genet. 2022 Nov 21;31(3):329–37. doi: 10.1038/s41431-022-01227-2 (PMC9995356; doi:10.1038/s41431-022-01227-2)
Supplement: Supplementary file 1 — Supplementary figure 1 [file 41431_2022_1227_MOESM1_ESM.pdf]

Supplementary figure 1. The figure shows the bioinformatic pipeline with software´s and different filters used in the study.

Bioinformatic pipeline	Description: software: filters
BCL	Raw sequencing data
LinkSeq Demux	Basecall/demultiplex: bcl2fastq v2.20
	3’end adapter trimming: BBtools v37.62
	Poly-G tails trimming: fastp v0.20
	Trimming bases with poor quality: Sickle v1.33
	Trimming reads with 10x Genomics barcodes: in-house developed algorithm.
FASTQ	Sequencing reads
LinkSeq Alignment	Aligning reads to GRCh38/hg38: EMA v0.6 aligner
	Aligning reads with no barcodes: BWA v0.7
	Marking duplicate reads, indexing, and Base Quality Score Recalibration: GATK v4.1
	Samples with average coverage under 5x were excluded.
BAM	Aligned reads
Variant discovery and annotation	Variant calling (GRCh38, dbSNP 138, SureSelectXT Human All Exon v6): GATK HaplotypeCaller
	Joint genotyping (quality score > 200): GATK GenotypeGVCFs.
	SNP filtering (6 Gaussians): GATK v4.1 Variant Quality Score Recalibration (VQSR).
	Indels filtering (4 Gaussians): GATK v4.1 Variant Quality Score Recalibration (VQSR).
	Genotype calls refinement: GATK v4.1 CalculateGenotypePosteriors,
	Removal of invariant sites and unused alternate alleles that did not pass QC.
	Variant annotation (dbSNP 138): GATK v4.1 VariantAnnotator and SnpEff v4.3
VCF	Multi-sample variant file
Variant exclusion filtering	Variants in the 50 to 100 VQSR tranches
	SNPs: QD < 2.0, SOR > 3.0, FS > 60.0, MQ < 40.0, MQRankSum < -12.5, ReadPosRankSum < -8.0, genotype quality < 20, HWE p < 1e-9
	Indels: QD < 2.0, FS > 200.0, ReadPosRankSum < -20.0, genotype quality < 40, HWE p < 1e-6
	Heterozygous genotypes with allelic balance less than 0.25
	Singletons were removed due to overall bad quality
	Heterozygote/homozygote rate >?
VCF	Filtered multi-sample variant file
